# Infection with *Hymenolepis diminuta* (Rodolphi, 1819) in a Child from North of Iran: Case Report

**Published:** 2019-08

**Authors:** Meysam SHARIFDINI, Elham HAJIALILO, Laleh GHANBARZADEH, Mehrzad SARAEI

**Affiliations:** 1. Department of Medical Parasitology and Mycology, School of Medicine, Guilan University of Medical Sciences, Rasht, Iran; 2. Department of Parasitology and Mycology, School of Medicine, Qazvin University of Medical Sciences, Qazvin, Iran; 3. Student Research Committee, Qazvin University of Medical Sciences, Qazvin, Iran; 4. Cellular and Molecular Research Center, Qazvin University of Medical Sciences, Qazvin, Iran

**Keywords:** *Hymenolepis diminuta*, Gastrointestinal symptoms, Praziquantel, Iran

## Abstract

We report a human case of *Hymenolepis diminuta* infection in Guilan Province, northern part of Iran in 2017. The patient was a 15-month-old boy with gastrointestinal symptoms. In stool examination, eggs of *H. diminuta* was found based on morphological characteristic. The infant was successfully treated with a single oral dose of praziquantel, and then completely recovered. For the first time, we report human infection with this species in north of Iran.

## Introduction

*Hymenolepis diminuta* is primarily a parasite of rodents throughout the world but it can rarely infect humans (1, 2). More than 20 different species of arthropods including rat and mouse fleas, flour beetles, cockroaches and caterpillars can serve as suitable intermediate hosts for the development of the cysticercoid larvae (2). Human infection with *H. diminuta* most often occurs from accidental ingestion of stored-grain beetles (*Tribolium* spp.) (1, 3). Adult worms attach themselves to the mucosa of the small intestine of human, and it passes eggs in the stool of patient (4). Despite, human infection is more common in children; it can be seen in every age group (5).

The rate of infection is different in various populations around the world. Human infection among children is common in a few countries such as New Guinea (1.9%), India (1.3%), Morocco (0.4%) and Turkey (0.05%–0.1%) (6), other children infection of the worm was reported from Italy, Malaysia and India (7–9). Despite some human cases reported from the northeast, south, and center (Persian Gulf area and Tehran) of Iran, there was not any case reported from northeast (Mashhad area) part of the country (10–12).

Here we report one case of *H. diminuta* infection in a child from Guilan Province, north of Iran.

## Case presentation

In Sep 2017, a 15-month-old boy from a rural area of Fouman region located in Guilan Province, northern part of Iran was referred to the Fouman Health Center of the Guilan University of Medical Sciences. The patient was suffering from abdominal pain, vomiting, nausea, anorexia, abdominal bloating, and mild diarrhea. Above all, he had lost weight as well as pale complexion. Laboratory result showed that the white blood cell count (WBC) of the patient was 12.200/mm^3^ with 23.2% neutrophils, 66.7% lymphocytes, 2% eosinophils, and 1% monocytes while the level of Hb was 11.5 g/dl.

**Fig. 1:**
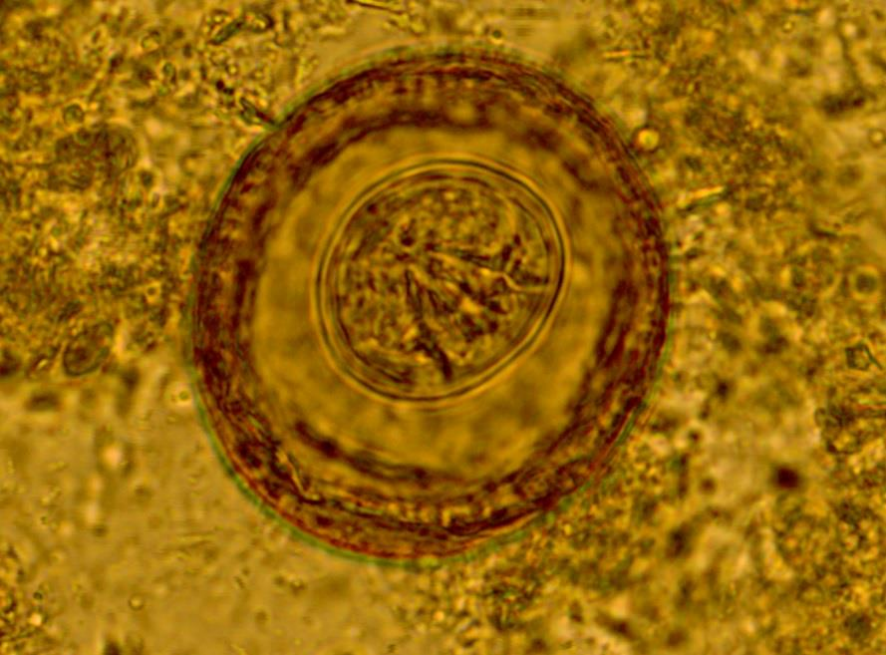
Light microscope view of *Hymenolepis diminuta* egg in the patient’s stool sample at ×400 magnification. The egg contains six central hooklets but no polar filaments (Original)

Stool parasitological examinations including direct smear and formalin-ether concentration techniques revealed that there were numerous numbers of eggs of *H. diminuta* in his stool sample (Fig. 1). The patient was treated with oral praziquantel (4.5 tablet 50 mg) in a single dose and finally, he turned asymptomatic. The infant was followed up by medical team and after two months of treatment, his stool sample was completely out of *H. diminuta* eggs.

Informed consent was taken from the parents of the patient before reporting the paper.

## Discussion

Human is accidental host of *H. diminuta* by ingestion of the intermediate hosts (5). Therefore, only a few hundred cases have been reported from different countries throughout the world (13–15, 7, 8). In Iran, *H. diminuta* is commonly found in rats and mice (16, 17). Until now, seven *H. diminuta* human infections have been reported in the country, and most of them affected children. The first reported case was a 10-year-old boy in Mashhad area in the northeast of Iran in 1968 (11). On the other hand, during an epidemiological survey in villages of Minab district in southern part of the country in 1972, five cases of infection were found among 635 examined individuals; including four children (9, 10, and 11 yr old boys and a 6 yr old girl) and one 65 yr old man (12). Moreover, the last case was reported in 2008, a 16-month-old female infant in Tehran (10). It was the first report of human infection of *H. diminuta* in the north of country, based on our knowledge. Most human infections to *H. diminuta* are asymptomatic; however, some infected patients may suffer from gastrointestinal symptoms such as abdominal pain, nausea, anorexia, and diarrhea (6). Our patient was successfully treated with praziquantel, which is the chosen drug to treat *H. diminuta* infection.

Local’s eating habits, for instance, live beetle ingestion in some areas such as southeast of Asia plays an important role in rate of public human infection by the worm, as some Asian countries reported of the human infection of the worm (8, 18). Contrary, Iranian eating habit does not contain such materials, thus people are only infected by accidental ingestion.

## Conclusion

In order to improve knowledge about human infection of *H. diminuta* such as transmission route, epidemiology, clinical presentation, and treatment protocol. We recommend that every rare case of human infection should be reported.

## Ethical considerations

Ethical issues (Including plagiarism, informed consent, misconduct, data fabrication and/or falsification, double publication and/or submission, redundancy, etc.) have been completely observed by the authors.
